# A Concise Review of Amyloidosis in Animals

**DOI:** 10.1155/2012/427296

**Published:** 2012-03-15

**Authors:** Moges Woldemeskel

**Affiliations:** Tifton Veterinary Diagnostic and Investigational Laboratory, Department of Pathology, College of Veterinary Medicine, The University of Georgia, 43 Brighton Road, Tifton, GA 31793, USA

## Abstract

Amyloidosis refers to a group of protein misfolding diseases characterized by deposition of a particular amyloid protein in various organs and tissues of animals and humans. Various types and clinical forms of amyloidosis, in which the pathology and pathogenesis is diverse depending upon the underlying causes and species affected, are reported in domestic and wild animals. The clinical findings are also quite variable consequent to the variation of the tissues and organs involved and the extent of functional disruption of the affected organs in various animal species. The affected organs may be enlarged and exhibit variable pallor grossly, or the amyloid deposit may be discernible only after microscopic examination of the affected tissues. Amyloid appears as a pale eosinophilic homogenous extracellular deposit in tissues. However, microscopic examination and Congo red staining with green birefringence under polarized light are needed to confirm amyloid and differentiate it from other apparently similar extracellular deposits such as collagen and fibrin. Identifying the type of amyloid deposit needs immunohistochemical staining, ultrastructural characterization of the amyloid fibril, and if feasible also genetic studies of the involved species for clinical and prognostic purposes. This paper provides a concise review of the occurrence of amyloidosis in domestic and wild animals.

## 1. Introduction

Amyloid is a pathologic proteinaceous substance deposited between cells in various tissues and organs of the body in a wide variety of clinical settings [1]. Although there are other components present in the deposit, the amyloid protein fibril is the main component of the amyloid substance. The deposit differs in protein composition depending upon the types of amyloidosis and the different clinical forms. Each clinical entity of amyloidosis may be manifested by a distinct clinical form with chemically specific amyloid fibril protein. This indicates that amyloid is a biochemically heterogeneous substance, although there are similarities in properties and staining characteristics.

### 1.1. Nomenclature and Classification of Amyloidosis

According to the WHO-IUIS Nomenclature Subcommittee [2] on the nomenclature of amyloid and amyloidosis, amyloid and amyloidosis are classified based on the amyloid fibril protein, followed by a designation of the fibril protein precursor. The capital letter A for amyloid is followed by the protein designation in abbreviated form. For example AL-amyloid refers to the amyloid derived from immunoglobulin light chain and may be seen in amyloidosis including idiopathic (primary) amyloidosis associated with myeloma or macroglobulinaemia [2]. The origin of the fibril in AL-amyloidosis is an immunoglobulin light chain or immunoglobulin heavy chain fragment [3]. The amyloid fibril protein in the immunoglobulin heavy chain has been given the designation AH [2]. AA-amyloid refers to the amyloid derived from serum A-amyloid protein (SAA), with apoSAA as its precursor protein. AA-amyloid is deposited in tissues as reactive (secondary) amyloidosis and familial amyloidosis. Furthermore, amyloid deposit is classified based on various precursor proteins identified in different types and forms of amyloidosis. For example A*β* and *β* protein precursor (*β*PP) are respectively identified as the amyloid fibril protein and its precursor in Alzheimer's disease. Islet amyloid polypeptide is a precursor for AIAPP amyloid protein deposited in pancreatic amyloidosis. Familial amyloidosis in humans could be due to mutations in fibrinogen, lysozyme, apolipoprotein AI, and transthyretin (with ATTR as amyloid protein). Human hereditary systemic amyloidosis with renal involvement, which is clinically indistinguishable from AL-amyloid deposit, is reported to have deposition of apolipoprotein AI (apoAI) or AII, lysozyme, or fibrinogen. Deposition of native transthyretin is also associated with senile systemic amyloidosis in elderly patients [2, 3].

Different types and clinical forms of amyloidosis are known based on the deposition in tissues and organs of various domestic and wild animals. Amyloidosis involving several tissues and organs throughout the body is referred to as systemic amyloidosis. Systemic amyloid deposition can be due to AL-amyloidosis, AA-amyloidosis, or familial amyloidosis. Amyloid substance may be confined at a given area in the body in the form of localized amyloidosis. Various forms of local amyloid deposits are known in animals and humans. This includes deposition of A*β* protein in Alzheimer's disease, AIAPP in pancreatic islet, AANF in atrial amyloid deposit with *β*PP, islet amyloid polypeptide, and atrial natriuretic factor as their respective precursors (Table 1) [2]. The pathogenesis, pathology, and clinical presentations of amyloidosis are protean consequent to the diverse underlying causes of its various forms, species of animals affected, and the severity of functional disruption in different tissues and organs involved. The diagnosis of amyloidosis requires histopathologic identification of amyloid deposits in the affected tissues. This is confirmed by Congo red staining and green birefringence under polarized light.

This paper provides a concise review of amyloidosis and its associated factors and clinical syndromes encompassing various forms of amyloidosis in domestic and wild animals and would be of importance as a reference for veterinary professionals and students.

## 2. Pathology and Pathogenesis

The pathology and pathogenesis of amyloidosis is extremely variable due to the multifarious underlying causes of its various forms in different species of animals. About 20–25 different types of proteins with the ability to aggregate, insolubilize, and deposit in tissue as amyloid have been identified [4, 5]. A review of the pathology underlying various protein foldings in domestic animals including amyloidosis is given by Gruys [5]. In animals, at least eight different amyloid precursors have been described [6]. The precursor proteins in amyloid fibrils may be amyloidogenic mutants as in some familial amyloidosis, whereas other precursors are normal wild-type proteins [5, 7]. The exact mechanisms through which the proteins are converted into amyloid fibrils in vivo are not well known [7].

The pathogenesis of amyloid deposition in AA- and AL-amyloidosis differs in character [8]. Amyloid results from abnormal folding of proteins which are deposited as fibrils in extracellular tissues and disrupt normal function. Misfolded proteins are often unstable and self-associate, ultimately leading to the formation of oligomers and fibrils deposited in tissues. Normally, misfolded proteins are degraded intracellularly in proteasomes or extracellularly by macrophages, which in amyloidosis fail to occur [1]. In AL-amyloidosis unstable monoclonal immunoglobulin light chains, produced by a plasma cell dyscrasia, lead to the formation and deposition of fibrils [9]. In AA-amyloidosis, there is increased level of SAA, which is common in the inflammatory states. However, increased SAA is not sufficient for the deposition of amyloid leading to amyloidosis. The SAA deposition in AA-amyloidosis would be due to defect in the degrading monocyte-derived enzymes or genetically determined structural abnormality in SAA molecule [1]. Although acute phase SAA is mainly synthesized by hepatocytes, extrahepatic SAA expression and production is reported in several species of animals and humans [10]. For example in mice SAA mRNAs were found to be expressed in the heart, kidney, lung, intestine, spleen, and peritoneal macrophages [11]. A definite role for extrahepatically formed SAA is still unclear. However, there is some evidence linking the extrahepatic SAA production to amyloid deposition, such as in a rat synapsin I-Sa a1 (SYNI) transgenic mouse model [12] and in the brown chicken joint after intra-articular injection with various agents [13].

Other factors and products including amyloid-enhancing factor (AEF), the isoform or proteolytic cleavage site of SAA, and glycosaminoglycans (GAGs) are involved in the development of amyloidogenesis [10]. The AEF, prepared from AA laden mouse liver consisting of proteins that are chemically identical to the AA molecule, was capable of accelerating amyloidosis even in femtomolar doses and retained its biologic activity over a considerable period [14].

A single amyloidogenic protein may result in multiple forms of amyloid fibrils depending on their induction conditions, and multiple mechanisms for amyloidogenesis are expected to operate as witnessed with their fibrillar polymorphism [15]. Although there are divers proteins associated with the formation of amyloid, they are all characterized by misfolded proteins which result in self-associated and unstable fibrils [16]. Protein fibril is the main component of the amyloid substance; however, there are other components present, the importance of which is yet not well established in the pathogenesis of amyloidosis. All forms of amyloid contain the pentraxin glycoprotein amyloid P-component (AP) that most probably is bound to the protein fibrils directly. The unique *β*-sheet fibril of amyloid is very resistant to physical agents and also gives the amyloid substance many of its characteristic properties, including affinity to the dye Congo red and green birefringence under polarized light after such staining [7].

## 3. AA-Amyloidosis in Animals

AA-amyloidosis is the most common form of amyloidosis in domestic animals. It is associated with chronic inflammatory or neoplastic diseases (nonimmunocyte dyscrasia), or it may be idiopathic, where no underlying disease is found [16, 17]. In this form of amyloidosis, the deposited amyloid A is derived from the acute phase reactant, serum amyloid-A (SAA) [5, 17, 18], which is an apolipoprotein of high-density lipoproteins (HDL), classes 2 and 3. It is formed mainly in the liver upon stimulation by proinflammatory cytokines [5] and normally plays a role in cholesterol transport [18] and as a chemoattractant [19] in the inflammatory processes. When the concentration of this molecule is increased, typically as a result of chronic inflammation, certain isoforms of SAA are partially cleaved into fragments that have an increased propensity to form fibrillar aggregates of amyloid deposited systemically, mainly in the kidney, liver, and spleen [20]. In systemic AA-amyloidosis, macrophages are activated and elaborate endogenous pyrogens IL-1 and IL-6, which stimulate hepatocytes to synthesize and secrete SAA. During an inflammatory reaction, SAA in the serum may increase several 100 times normal concentrations. However, not all systemic inflammatory reactions lead to the formation of AA-amyloid, but only some of the inflammatory reactions lead to amyloidosis. Deposition of insoluble amyloid fibrils occurs either due to defect in SAA-degrading enzyme in the system, or consequent to the synthesis of abnormal SAA protein resistant to the enzymatic degradation [1, 16].

AA-amyloidosis is the most common type of amyloidosis in mammals and birds and often results in hepatic or renal failure due to physical disruption of the normal cellular and organ processes [21]. It is a common disease of water fowl and is characterized by the deposition of extracellular fibrils of amyloid-A protein in the liver and certain other organs in this species [22]. AA-amyloidosis is also reported in a wide variety of domestic animal species including canines, equines, bovines, avian species, porcines, felines, sheep, and goats [6, 23–30]. It is described in association with different chronic diseases in captive cheetah (*Acinonyx jubatus*), Siberian tigers (*Panthera tigris altaica*), mink (*Mustela vison*), black-footed cats (*Felis nigripes*), black-footed ferrets (*Mustela nigripes*), Dorcas gazelle (*Gazella dorcas*), mountain gazelle (*Gazella gazella*), bighorn and Dall's sheep, free-living lioness, and in swans and other anatidae (*Panthera leo*) [21, 31–41]. In the cheetahs, it is reported in association with chronic lymphoplasmacytic gastritis and as idiopathic in the Siberian tigers. In the cheetahs and the Siberian tigers, the deposits were primarily in the medullary interstitium, with minimal glomerular involvement [38, 39]. Deposition of the amyloid in renal amyloidosis reported in the Dorcas gazelle was also mainly in the renal medulla, sparing the glomeruli [35].

### 3.1. Familial Forms of AA-Amyloid Deposit in Animals

Familial amyloidosis refers to the deposition of amyloid protein in tissues of animals in a given genetically associated family prone to the deposition of amyloid fibrils. Some species of animals appear to have genetic predisposition to the deposition of AA-amyloidosis. In veterinary medicine, a few animal species are reported to be genetically prone to deposition of amyloid in various tissues. Familial amyloidosis is reported in Siamese and Abyssinian cats and Shar Pei dogs with the AA-proteins differing in primary sequences and patterns of deposition [23, 42, 43]. The kidney is the main target organ for the deposition of amyloid in the Abyssinian cat and Shar Pei dogs, while the amyloid protein is mainly deposited in the liver in Siamese cats [23, 43, 44]. Furthermore, analysis of SAA cDNA sequences from several animals identified a distinct genetic dimorphism that may be relevant to the susceptibility to secondary amyloid disease. The duck genome contained a single copy of the SAA gene that was expressed in the liver and lungs of ducklings, even in the absence of induction of acute phase response [22]. Amyloid-resistant mouse strains were found to have a non-amyloidogenic acute phase SAA [45, 46], and rats were shown not to form acute phase SAA and AA-amyloid at all [47, 48]. Additionally, there are other animal species that appear to be prone to amyloid deposition. The high prevalence of amyloidosis in captive cheetahs is suggested to indicate some level of familial predisposition similar to the Abyssinian cats [38]. Amyloid arthropathy frequently occurred in brown layer chickens, but never in white layers. The suspected higher susceptibility of brown layers was confirmed experimentally by inducing amyloidosis with an arthropathic and amyloidogenic strain of *E. faecalis *[49]. In the systemic amyloidosis reported in the black-footed cats, there was no association with concurrent chronic inflammatory conditions, indicating that the amyloid deposit was not secondary to inflammation. Small amounts of amyloid deposition were also detected in tissues from a single free-ranging black-footed cat indicating a predilection for amyloidosis in these cats. Moreover, heritability estimation also suggested that amyloidosis might be familial in this species [21]. Therefore, in addition to Siamese and Abyssinian cats and Shar Pei dogs, in which familial amyloidosis is well recognized in veterinary medicine, certain species such as captive cheetahs, Dorcas gazelles, black-footed cat, and brown layer chickens appear to be genetically predisposed to amyloidosis.

## 4. AL-Amyloidosis in Animals

AL-amyloidosis is very rare in domestic animals [17], unlike in humans, in which it is a common form of systemic amyloidosis. Report on systemic AL-amyloidosis in domestic animals is very rare. It is reported in a horse gelding with multiple myeloma [17], a mare [50], and in a cow with bovine leukocyte adhesion deficiency [51]. Local deposition of AL-amyloid is reported in various species of animals. This includes diffuse to nodular, tracheal, and bronchial AL-amyloidosis in dogs [52, 53] and cutaneous nodular amyloidosis in equines [54]. Recently, non-AA-amyloid is reported in two felines with thymomas [55]. AL-amyloid deposition was reported in cats and dogs in association with diffuse and local extramedullary plasmacytomas [56]. In animals, the deposition of AL-amyloid protein is generated following overproduction of monoclonal light chains associated with immunocyte dyscrasia. In this form of amyloidosis, plasma cells produce excessive quantities of immunoglobulin light chains that are resistant to complete enzymatic degradation and are susceptible to forming insoluble fibrils. The most common immunocyte dyscrasia associated with AL-amyloidosis in domestic animals is a neoplasm of plasma cell. The AL form of amyloid can contain complete immunoglobulin light chains, the NH_2_-terminus portion of immunoglobulin light chains, or both. Immunoglobulin secreting cells, B lymphocytes, and plasma cells are associated with the deposition of AL-amyloid [16].

In humans, the AL-amyloid type derived from immunoglobulin light chains is the most common form of systemic amyloidosis [57]. Small-sized bone marrow plasma cell clones are reported to produce toxic light chains that cause fibrillar deposits in multiple organs [58]. The amyloid fibrils are formed by the N-terminal fragment of a monoclonal immunoglobulin light chain comprising the variable region and a portion of the constant region. Only a small proportion of free monoclonal light chains form amyloid fibrils in vivo. Thus, the ability to form amyloid is probably related to individual structural characteristics of the light chain variable region. Unlike most other plasma cell dyscrasias, the *λ* light chain isotype is prevalent in AL (*κ*/*λ* ratio, 1 : 3), suggesting the existence of amyloid-associated V*λ* germ line genes [58]. In contrast to amyloidosis in humans, in which the majority of patients with AL do not have any overt B lymphocyte or plasma cell neoplasm, but do have monoclonal antibodies or light chains in their serum or urine, domestic species rarely have AL-type amyloid without evidence of an immune dyscrasia [16].

## 5. Localized and Other Forms of Amyloidosis in Animals

Localized amyloidosis refers to the deposition of amyloid fibrillar protein as a grossly visible mass or a microscopic deposit at a given site in an organ or tissue. Localized AL-amyloidosis is characterized by limited growth of monoclonal plasma cells and restriction of amyloid deposits to sites adjacent to those of the synthesis of the precursor [58]. Localized amyloidosis is reported in several animal species involving different tissues and organs. It is present in calcifying epithelial odontogenic tumors (amyloid producing odontogenic tumors) of the cat and dog and pancreatic islets in cats [5, 16]. A*β*-amyloid and APrPsc-amyloid can be encountered in the brains of old dogs and sheep with scrapie, respectively [5, 59]. Some forms of naturally occurring transmissible spongiform encephalopathies such as chronic wasting diseases (CWD) are characterized by amyloid plaques in the brain in addition to the intraneural vacuolation [16]. Deposition of amyloid derived from islet amyloid polypeptide (IAPP), a normal protein secreted by the *β* cell of the pancreas, is reported in the pancreas of cats and macaques. The mechanisms by which IAPP undergoes assembly and transformation into deposits of amyloid fibrils are not fully understood [60, 61]. However, the islet amyloid deposits in humans and feline and macaque animal models of type 2 diabetes mellitus are associated with significant loss of islet *β* cells [61]. It is not known if the deposition of amyloid and the development of clinical diabetes mellitus is the result of progressive loss of *β* cells from the amyloid deposit or if the deposition of the amyloid occurs as a result of prolonged stimulation of the *β* cells as a consequence of insulin resistance [16].

In horses, localized AL-amyloidosis is described in the skin as cutaneous amyloidosis associated with lymphoma [62] and extramedullary plasmacytoma [63]. Hepatic AA-amyloidosis is commonly reported in serum-producing horses [64].

In dogs, cutaneous amyloidosis can occur as primary localized form or secondary to systemic amyloidosis. Most cutaneous amyloidoses in dogs are suggested to be of immunoglobulin light chain (AL) in type [65–67] and are reported in association with localized plasma cell proliferation, plasma cell dyscrasia, or cutaneous extramedullary plasmacytoma [65, 68, 69]. As part of primary systemic disease, diffuse, finely distributed cutaneous deposition is reported in dogs with monoclonal gammopathy [66, 67, 69] and dermatomyositis [69]. However, primary localized cutaneous form, which is rare in dogs [65, 66, 69], shows solitary or rarely group of nodules in the subcutis and dermis. The ears are most commonly involved [66, 67] although lesions would be seen at any site on the body [66].

Similar to human senile amyloidosis, old dogs develop neurodegenerative brain changes including cerebrovascular amyloidosis and senile plaques with amyloid deposition. The amyloid protein in the canine cerebrovascular amyloidosis is deposited in the medium- and small-caliber arterioles and capillaries of the leptomeninges and the brain parenchyma. Vascular or perivascular degeneration or cellular reactions were not detected in affected vessels [70]. Such amyloid deposits in the brain consist of a number of extracellular proteins, but most commonly contain A*β* type amyloid, which consists of a proteolytic fragment of the APP (Amyloid Precursor Protein). The A*β* form of amyloid is associated with the cerebral amyloid angiopathy of Alzheimer's disease in humans and with neurodegeneration in the canine brain [16]. In the brain tissue of aged dogs, Alzheimer-like pathology with lipofuscin being present in neurons and macrophages, A*β*-precursor protein in neurons, A*β*-positive plaques, and 4-hydroxynonenal in neurons and macrophages, and limited intraneuronal accumulation of tau and advanced glycation end products increasing with longevity, has been encountered [71–74]. The amyloidosis which causes neurodegenerative disorders is almost always related to the intracerebral production of the pathogenic protein since most proteins, like immunoglobulins, do not cross the blood brain-barrier. One exception may be the case of systemic amyloidosis related to transthyretin mutations, which produce peripheral neuropathy and cerebral changes in the white matter in some cases [75] and hematogenic pattern of vascular deposition following systemic amyloidosis in areas of the brain that lack tight blood-brain barrier such as small circumventricular areas around the third and fourth ventricles, the infundibulum, and area postrema [76]. A*β*-amyloid in the brain tissue of aged dogs, showing signs of dementia, forms a canine counterpart of senile dementia of the Alzheimer type (ccSDAT) in man. Other organ systems containing amyloid in the aged dog are the heart, gastrointestinal tract, and lungs. The deposition of amyloid in the pulmonary vasculature of the aged dogs was reported to be derived from apolipoprotein AI (Apo AI) [16]. Severe senile amyloidosis is also reported to be a characteristic of age-associated disorder in the SAMP1 and SAMP10 strains of mice, making them a valuable model to investigate pathogenesis of amyloidosis and to assist in the development of effective therapeutic modalities [77].

## 6. Clinical Findings

Amyloidosis is a feature of several different pathologic mechanisms and as such should not be considered a single disease, but rather a group of diseases having in common the deposition of similar appearing proteins [16]. Animals affected with amyloidosis may show variable clinical signs due to variation in the main underlying disease as commonly seen in AA-amyloidosis or those solely attributable to the deposition of amyloid in a given tissue or both. Some local amyloid depositions may be clinically nonsignificant incidental findings in certain tissues. In most cases, small local amyloid masses pose no clinical problems. Therefore, the clinical finding of amyloidosis in affected animals is variable and reflects the extent of perturbed function of the predominantly affected organs and tissues due to the deposition of amyloid or may show variable clinical signs that may be associated with the underlying chronic disease and the concurrent amyloid deposit. For example, kidney is the main target organ for the deposition of amyloid in familial amyloidosis of the Abyssinian cat (glomerular deposit) and Shar Pei dogs (medullary deposit), while the amyloid is mainly deposited in the liver in Siamese cats [23, 44]. Because amyloid deposit in the amyloidosis of the Abyssinian cat and Shar Pei dog is primarily a renal deposit, clinical signs associated with disrupted renal function will be seen in the affected Abyssinian cats and Shar Pei dogs, whereas clinical signs associated with derailed hepatic function may be manifested in Siamese cats. Deposition of amyloid in the pancreas of cats, nonhuman primates (macaques and baboons), and humans can lead to the development of type 2 diabetes mellitus [16, 61]. Amyloid deposition can also occur in the cortex of the adrenal gland; however, it is not associated with any functional deficiencies [16].

Reactive systemic amyloidosis secondary to chronic inflammatory conditions is often the most severe of the systemic forms. Liver, kidneys, spleen, lymph nodes, and adrenal glands are most commonly affected. Animals with renal amyloidosis frequently die from renal failure [16]. The clinical sign usually reflects the functional disruption and severity of the particular site of the kidney affected in renal amyloidosis. Progressive renal failure was the cause of death in Dorcas gazelles with renal medullary amyloidosis [35]. In contrast, although renal medullary amyloidosis occurs in most cases of bovine renal amyloidosis, it is a subclinical disease less hazardous to renal function and thus less important in clinical disease than glomerular deposition [78]. In Dorcas gazelle impairment of the renal function involved obstruction of the medullary tubules and collecting ducts, leading to atrophy and eventual loss of nephrons, with progressive interstitial fibrosis in the medulla and cortex [35]. Interstitial amyloidosis may also contribute to loss of concentrating ability and renal failure by interfering directly with maintenance of the medullary interstitial concentration gradient necessary for reabsorptive function in the renal tubules [38]. In 74% of the cheetahs with systemic amyloidosis associated with chronic gastritis, renal failure was determined to be the sole or partial cause of death [38]. Clinical signs including rapid weight loss, muscle atrophy, soft unformed stool, and ventral edema were noted in a case of a horse gelding with AL amyloidosis associated with multiple myeloma [17].

## 7. Gross and Microscopic Lesions

Several different pathologic mechanisms and conditions underlie various forms and types of amyloidosis although abnormal proteins with similar staining characteristics are deposited in various organs and tissues of the affected animals. The gross feature of amyloidosis due to the deposited amyloid in these tissues and organs is not specific for amyloid. The affected organs are often enlarged, moderately firm, and abnormally discolored [16]. In AA-amyloidosis, the deposition in most species is in the central organs and tissues such as spleen, liver, kidney, and the arterial walls [20, 79]. Experimentally induced AA Amyloidosis in sheep principally affected the gastrointestinal tract [80], and scattered amyloid deposits without clinical diabetes are usually observed in feline pancreas in increasing age [81]. Depending on the extent of the deposition, there may be splenomegaly, hepatomegaly, and renomegaly as spleen, liver, and kidneys are the most commonly affected organs in systemic AA-amyloidosis.

**Figure 1:**
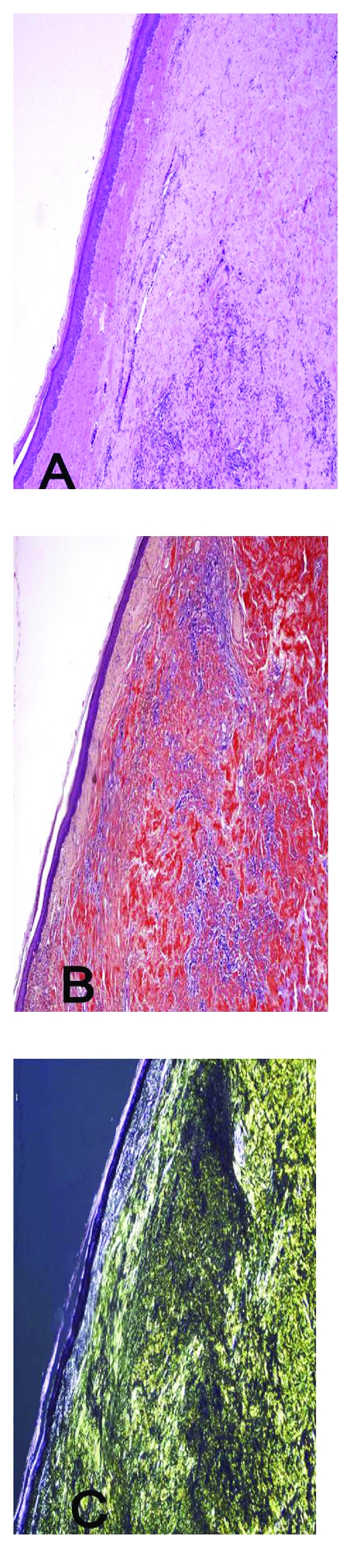
Histological features of skin of a dog with nodular cutaneous amyloidosis, which stained light eosinophilic with haematoxylin-eosin (A) and red with Congo red stain (B). The amyloid showed yellow-green birefringence (C) illuminated with polarized light, characteristic of amyloid deposits.

Microscopic findings of the deposition of amyloid protein may correspond to the grossly visible lesions, or it may be seen only after microscopic examination without distinct grossly discernible lesions. Amyloid should be differentiated from other similar-appearing extracellular deposits such as collagen and fibrin [16]. For a fibrillary protein to be considered amyloidogenic it should produce extracellular deposits with affinity for the Congo red dye and a green birefringence under polarized light (Figure 1).

Congo red stain, which does not have chemical specificity for amyloid, but is dependent on the conformational property of being arranged in beta-pleated sheets, is the most commonly used stain for the identification of amyloid. Amyloid stains orange to red under light microscopy, which appear as an apple-green birefringent material under polarized light [6, 16]. Immunohistochemistry can also be used not only to identify amyloid deposits but also to identify the specific constituents composing the deposits such as the anti-*β*-light chain antibodies [16]. AA- and AL-amyloid deposits stain similar with Congo red stain. The identification of AA is usually based on its reactivity with specific anti-AA antibodies and sensitivity to permanganate pretreatment [82, 83]. The common ultrastructure of amyloid proteins is made of some nonbranching, rigid fibrils, 7.5 to 10 nm wide, and of variable length, which arrange themselves in antiparallel sheets with *β* structure [75].

Microscopically, amyloid is deposited extracellularly in various affected tissues. The involved tissues and organs vary in different animal species and types and forms of amyloidosis. Amyloid deposition was seen in the kidneys, blood vessels, spleen, liver, lymph nodes, gastrointestinal tract, and adrenal glands of sheep and goats with AA amyloidosis [6]. There was widening of the medullary interstitium by eosinophilic homogeneous material, which encroached upon medullary tubules and collecting ducts with occasional renal papillary necrosis in Dorcas gazelle with medullary amyloidosis. The amyloid deposition significantly correlated with interstitial fibrosis, and tubular dilation and atrophy [35].

In a horse with AL-amyloidosis associated with multiple myeloma, diffuse severe extracellular amyloid deposits were present in the lamina propria of glandular stomach, duodenum, and jejunum. Much of the spleen and sternal bone marrow were replaced by neoplastic round cells, and multiple foci of amyloid were also present in the spleen and bone marrow. No significant microscopic changes were noted in the kidneys, liver, and lungs [17]. In a cow with systemic AL-amyloidosis associated with bovine leukocyte adhesion deficiency, amyloid deposits immunohistochemically related to immunoglobulin kappa-light chains of precursor protein were present in the perivascular and intercellular spaces of the visceral organs, such as the liver, kidneys, pancreas, adrenal glands, and upper alimentary tract [51].

## 8. Infectivity of Prion Diseases and Amyloid Proteins

Proteins are a major constituent of cells with specific biological functions. Besides the primary structure of the sequence of amino acids that comprise a protein, the secondary structure represents the first step of folding, defining the general conformation of proteins. The biological functions of proteins are directly dependent on the acquisition of their conformation [84]. Protein misfolding, which is central to the pathogenesis of several neurodegenerative disorders [85], is caused by deposition of misfolded proteins (prions). Prion diseases are protein misfolding disorders in which misfolding of a host-encoded prion protein (PrP) occurs. PrP may exist as a normal cellular prion protein designated as PrPC and a pathogenic misfolded conformer designated as PrPSc. The pathogenesis of prion diseases is associated with the accumulation of the aggregates of misfolded conformers (PrPSc) [86]. PrPSc are infectious, naturally transmissible misfolded proteins with neurotoxic properties and cause fatal neurological diseases in humans and wide range of animal species [85–87].

Sixteen different variants of prion disease have been identified in humans and animals [86]. Animal prion diseases include scrapie of sheep and goats, bovine spongiform encephalopathy (BSE) or mad cow disease, transmissible mink encephalopathy, feline spongiform encephalopathy, exotic ungulate spongiform encephalopathy, chronic wasting disease of cervids, and spongiform encephalopathy of primates [86]. In humans, prion diseases are traditionally classified into Creutzfeldt-Jakob disease (CJD), its variant form (vCJD) resulting from human infection by BSE prions, Gerstmann-Sträussler-Scheinker disease (GSS) and fatal familial insomnia (FFI) [85].

Prion diseases can arise without any apparent cause as in sporadic Creutzfeldt-Jakob disease (CJD) or due to genetic disorders linked to mutations in the endogenous PrP protein. The diseases can also be acquired by infection, through ingestion of contaminated products or through iatrogenic procedures and are in most cases experimentally transmissible [86]. Infectivity and transmissibility of prion disease has been much of a concern particularly since the outbreak of BSE in cattle. Cattle are infected with BSE when they ingest prion-contaminated meat and bone meal of ruminant origin contaminated with prions. Consumption of BSE-contaminated bovine tissues is associated with a fatal variant form of Creutzfeldt-Jakob disease (vCJD) in humans [87, 88]. The variant form of Creutzfeldt-Jakob disease can also be transmitted from humans to humans by blood transfusion [89]. Transmission of sheep scrapie agent through secretion of prions into milk from ewe with scrapie and lentiviral mastitis is reported to infect 90% of naïve suckling lambs [90]. Goats can be infected by contact with scrapie-infected sheep, either directly or by exposure to contaminated pastures [87].

Prion diseases and amyloidosis present many similar aspects of the so-called conformational diseases, according to the interpretation which prion infections could be considered as a form of transmissible cerebral amyloidosis. This emerging concept of prion infections as a form of transmissible cerebral amyloidosis is an application of the prions' transmissibility concept to a broader spectrum of amyloid. Amyloid fibers show an extremely high content of *β*-sheet, in a very similar structure to the one observed among prion rods, associated to the transmissible spongiform encephalopathies. Amyloid diseases such as Alzheimer's and Parkinson's disease are “infectious” in the sense that misfolded *β*-sheeted conformers formed in a nucleation process in which preformed metastable oligomer acts as a seed to convert a normal isoform into an abnormal protein with a misfolded conformation [84]. Although only prion infections have a proven infectivity in a microbiological sense [84], Lundmark et al. [14] detected the transmissibility of systemic amyloidosis by a prion-like mechanism among mice. This transmissibility of systemic amyloidosis by a prion-like mechanism in a very effective way provided evidence that some forms of amyloidosis are transmissible diseases, similar to the prion-associated disorders [14].

As described earlier, prions in different types of transmissible spongiform encephalopathies and amyloidosis are demonstrated to be naturally and experimentally transmissible. Furthermore, prions are reported to be more resistant than endospores to standard inactivation methods such as heat, irradiation, and chemicals [88]. This necessitates establishing reliable diagnostic procedures to avoid transmission of prion proteins from animals to humans through consumption of infected or contaminated animal products.

Some efforts are made to develop rapid detection of abnormal proteins in animal tissues. SAA level was shown to increase in cows with pathological conditions as compared to healthy cattle. Although it did not reveal a clear association between acute phase proteins levels and respective specific pathological conditions, determination of the serum levels of the acute phase proteins such as SAA could be a useful tool enabling separation of “suspect” from “nonsuspect” animals before transport to slaughter or during antemortem inspection in meat inspection systems [91]. However, because high blood levels of the acute phase proteins are temporary, can be associated with various conditions, may subside later, and lack standardization, acute phase protein analysis alone is not reliable and should be used in association with other tools of inspection [91]. Therefore, it is necessary to develop other rapid and accurate method of antemortem or post-mortem diagnosis of abnormal proteins, as part of meat inspection procedure. In Japan, PCR identification of species-specific, animal group-specific, and plant DNA is employed as part of feed ban in place for the control of BSE. The PCR method is of fundamental importance for the detection of prohibited animal-derived material in feed [92] and could be employed as part of inspection of meat and other products of animal origin intended for human consumption.

## 9. Conclusion

In summary, the pathology and clinical findings of amyloidosis in animals are diverse depending upon the underlying causes and species affected. The functional derangement of the tissues and organs involved and the extent of functional disruption of the affected organs in various animal species as well as concurrent infections influence the various clinical presentations seen in animals with amyloidosis.

Microscopic examination and Congo red staining with green birefringence under polarized light are required to confirm deposition of amyloid substance and thereby rule out similar extracellular deposits such as collagen and fibrin. Although this is used as a diagnostic criteria for amyloid deposits, identifying the type of amyloid deposited, differentiating local from systemic (primary or secondary), or inherited familial deposits from acquired conditions, would be important for clinical management of the patient. Therefore, apart from confirmation of amyloid with Congo red stain, classification using immunohistochemical staining, ultrastructural characterization of the amyloid fibril, and if feasible genetic studies of the involved species should be considered for clinical and prognostic purposes.

## Figures and Tables

**Table 1:** Nomenclature and classification of amyloid and amyloidosis. Source: WHO/IUIS Nomenclature subcommittee [2].

Amyloid protein^a, b^	Protein precursor	Protein type	Clinical diagnosis
AA	apoSAA		Reactive (secondary) amyloidosis, familial mediterranean fever, familial amyloid nephropathy with urticarial and deafness (Muckie-Wells' syndrome)
AL	*κλ*, for example, *κ*III	A*κ*, A*λ*, for example, A*κ*III	Idiopathic (primary) amyloidosis associated with myeloma/macroglobulinaemia
AH	IgG1 (*γ* ^1^)	A*γ* ^1^	
ATTR	Transthyretin	For example, Met 30^c^ For example, Met III TTR or IIe 122	Familial amyloid polyneuropathy, Portuguese Familial amyloid cardiomyopathy, Danish Systemic senile amyloidosis
AApoAI	apoAI	Arg 26	Familial amyloid polyneuropathy, Iowa
AGel	Gelsolin	Asn 187^d^ (15)	Familial amyloidosis, Finish
ACys	Cystatin C	Gin 68	Hereditary cerebral hemorrhage with amyloidosis, Icelandic
A*β*	*β* protein precursor for example, *β*PP 695^e^	Gin 618 (22)	Alzheimer's disease, Down syndrome, and hereditary cerebral hemorrhage with amyloidosis, Dutch
A*β* 2M	*β*2-microglobulin		Associated with chronic dialysis
AScr	Scrapie protein precursor 33–35^f^ cellular form	Scrapie protein 27–30	Creutzfeldt-Jakob disease, and so forth
		For example, Leu 102	Gerstmann-Straüssler-Scheinker syndrome
ACal	(Pro)calcitonin	(Pro)calcitonin	In medullary carcinomas of the thyroid
AANF	Atrial natriuretic factor		Isolated atrial amyloid
AIAPP	Islet amyloid polypeptide		In islets of Langerhans, diabetes type II, insulinoma
AIns^g^	Insulin		Islet amyloid in the degu (a rodent)
AApoAII^g^	apoAII (murine)	Gin5	Amyloidosis in senescence, accelerated mice

^a^Nonfibrillar proteins, for example, protein AP (amyloid P-component) excluded.

^b^AA: amyloid A protein; SAA: serum amyloid A protein; apo: apolipoprotein; L: immunoglobulin light chain; H: immunoglobulin heavy chain.

^c^ATTR Met 30 when used in text.

^d^Amino acid position in the mature precursor protein. The position in the amyloid fibril protein is given in parentheses.

^e^Number of amino acid residues; ^f^Molecular mass (kilodaltons); ^g^Not found in humans.
